# PSMA-PET/CT-guided salvage radiotherapy in recurrent or persistent prostate cancer and PSA < 0.2 ng/ml

**DOI:** 10.1007/s00259-023-06185-5

**Published:** 2023-03-11

**Authors:** Nantia Solomonidou, Daphnie Germanou, Iosif Strouthos, Efstratios Karagiannis, Andrea Farolfi, Stefan A. Koerber, Juergen Debus, Jan C. Peeken, Marco E. Vogel, Alexis Vrachimis, Simon K. B. Spohn, Mohamed Shelan, Daniel Aebersold, Anca-Ligia Grosu, Francesco Ceci, Stephanie G. C. Kroeze, Matthias Guckenberger, Stefano Fanti, Claus Belka, George Hruby, S. Scharl, Thomas Wiegel, Peter Bartenstein, Christoph Henkenberens, Louise Emmett, Nina Sophie Schmidt-Hegemann, Konstantinos Ferentinos, Constantinos Zamboglou

**Affiliations:** 1 Department of Radiation Oncology, German Oncology Center, University Hospital of the European University, Limassol, Cyprus; 2 Division of Nuclear Medicine, IRCCS Azienda Ospedaliero–Universitaria di Bologna, Bologna, Italy; 3 Department of Radiation Oncology, Heidelberg University Hospital, Heidelberg, Germany; 4 Clinical Cooperation Unit Radiation Oncology, German Cancer Research Center, Heidelberg, Germany; 5 Department of Radiation Oncology, Klinikum rechts der Isar, Technical University of Munich (TUM), Munich, Germany; 6 Department of Radiation Sciences (DRS), Institute of Radiation Medicine (IRM), Helmholtz Zentrum, Munich, Germany; 7 Deutsches Konsortium für Translationale Krebsforschung (DKTK), Partner Site Munich, Munich, Germany; 8 Department of Nuclear Medicine, German Oncology Center, University Hospital of the European University, Limassol, Cyprus; 9 C.A.R.I.C. Cancer Research & Innovation Center, Limassol, Cyprus; 10 Department of Radiation Oncology, Medical Center – University of Freiburg, Faculty of Medicine, University of Freiburg, Freiburg im Breisgau, Germany; 11 German Cancer Consortium (DKTK), Partner Site Freiburg, Freiburg im Breisgau, Germany; 12 Berta-Ottenstein-Programme, Faculty of Medicine, University of Freiburg, Freiburg im Breisgau, Germany; 13 Department of Radiation Oncology, Inselspital Bern, University of Bern, Bern, Switzerland; 14 Division of Nuclear Medicine, IEO European Institute of Oncology IRCCS, Milan, Italy; 15 Department of Oncology and Hemato-Oncology, University of Milan, Milan, Italy; 16 Northern Sydney Cancer Centre, Royal North Shore Hospital, University of Sydney, Sydney, New South Wales Australia; 17 Department of Radiation Oncology, University Hospital, LMU Munich, Munich, Germany; 18 Department of Radiation Oncology, University of Ulm, Ulm, Germany; 19 Department of Nuclear MedicineUniversity Hospital, LMU Munich, Munich, Germany; 20 Department of Radiotherapy and Special Oncology, Medical School Hannover, Hannover, Germany; 21 Department of Theranostics and Nuclear Medicine, St Vincent’s Hospital, Sydney, Australia; 22 St Vincent’s Clinical School, University of New South Wales, Sydney, Australia; 23 German Oncology Center, University Hospital of the European University Cyprus, Limassol, Cyprus

**Keywords:** PSMA-PET, Prostate cancer, Salvage radiotherapy, Prostate-specific antigen

## Abstract

**Purpose:**

The purpose of this retrospective, multicenter study was to assess efficacy of PSMA-PET/CT-guided salvage radiotherapy (sRT) in patients with recurrent or persistent PSA after primary surgery and PSA levels < 0.2 ng/ml.

**Methods:**

The study included patients from a pooled cohort (*n* = 1223) of 11 centers from 6 countries. Patients with PSA levels > 0.2 ng/ml prior to sRT or without sRT to the prostatic fossa were excluded. The primary study endpoint was biochemical recurrence-free survival (BRFS) and BR was defined as PSA nadir after sRT + 0.2 ng/ml. Cox regression analysis was performed to assess the impact of clinical parameters on BRFS. Recurrence patterns after sRT were analyzed.

**Results:**

The final cohort consisted of 273 patients; 78/273 (28.6%) and 48/273 (17.6%) patients had local or nodal recurrence on PET/CT. The most frequently applied sRT dose to the prostatic fossa was 66–70 Gy (*n* = 143/273, 52.4%). SRT to pelvic lymphatics was delivered in 87/273 (31.9%) patients and androgen deprivation therapy was given to 36/273 (13.2%) patients. After a median follow-up time of 31.1 months (IQR: 20–44), 60/273 (22%) patients had biochemical recurrence. The 2- and 3-year BRFS was 90.1% and 79.2%, respectively. The presence of seminal vesicle invasion in surgery (*p* = 0.019) and local recurrences in PET/CT (*p* = 0.039) had a significant impact on BR in multivariate analysis. In 16 patients, information on recurrence patterns on PSMA-PET/CT after sRT was available and one had recurrent disease inside the RT field.

**Conclusion:**

This multicenter analysis suggests that implementation of PSMA-PET/CT imaging for sRT guidance might be of benefit for patients with very low PSA levels after surgery due to promising BRFS rates and a low number of relapses within the sRT field.

**Supplementary information:**

The online version contains supplementary material available at 10.1007/s00259-023-06185-5.

## Introduction

Biochemical recurrence (BR) post-radical prostatectomy (RP) occurs in approximately 30% of prostate cancer patients [1]. Before the incorporation of prostate-specific membrane antigen positron emission tomography/computed tomography (PSMA-PET/CT) imaging for salvage radiotherapy (sRT) guidance, several studies reported favorable metastasis-free survival (MFS) rates for the conduction of sRT at very low prostate-specific antigen (PSA) levels (< 0.2 ng/ml) [2, 3]. Furthermore, the prospective randomized controlled RADICALS trial compared adjuvant radiotherapy with very early sRT [4]. The authors included patients for very early sRT after three consecutive PSA rises or at PSA > 0.1 ng/ml and two consecutive rises. Consequently, current national comprehensive cancer network guidelines (NCCNv2023.1) recommend the conduction of very early sRT at PSA levels > 0.1 ng/ml or at two consecutive rises.

Recently, PSMA-PET/CT has been incorporated as a sensitive tool to detect recurrent lesions after prostatectomy. The detection rate of PSMA-PET/CT positive lesions increases at higher PSA serum levels. For example, in a prospective analysis including 635 patients, the detection rates were 97%, 86%, 84%, 57%, and 38% for PSA levels of ≥ 5, 2 to < 5, 1 to < 2, 0.5 to < 1, and 0.2 to < 0.5 ng/ml, respectively [5]. PSMA-PET/CT is not routinely used for staging patients with BR at low PSA serum levels (< 0.2 ng/ml). However, two recent prospective studies including patients with PSA < 0.2 ng/ml reported a detection rate of 51.2% [6] and 44.8% [7], respectively.

Regarding the discrepancy between low PSMA-PET/CT detection levels and favorable sRT results, there is no consensus, at present, about the role of PSMA-PET/CT imaging in patients with BR and PSA levels < 0.2 ng/ml after RP. Thus, the main aim was to assess the outcome after PSMA-PET/CT-based sRT in terms of biochemical recurrence-free survival (BRFS). In addition, univariate and multivariate Cox regression analyses have been used to assess the impact of several risk factors on PSA relapse. Third, recurrence patterns after sRT have been described.

## Materials and methods

### Patients

Patient data for this retrospective study was extracted and pooled for analysis from 11 medical centers from 6 countries and local ethics committees from all institutions gave their approval. The inclusion criteria for the pooled database (*n* = 1223) consisted of patients who underwent open or laparoscopic RP and subsequently received PSMA-PET/CT-based sRT for a PSA persistence or recurrence (PSA after prostatectomy ≥ 0.1 ng/ml for both). To create the subgroup for this analysis, patients who had PSA > 0.2 ng/ml (*n* = 942), who did not receive RT to all PET positive lesions (*n* = 2) and RT was not performed to the prostatic fossa (*n* = 4), were excluded. Finally, 273 patients met the inclusion criteria**.**

### PSMA-PET/CT/CT scans prior to sRT

PET/CT prior to sRT were performed with ^68^ Ga-PSMA-11 (*n* = 224), ^68^ Ga-PSMA-I&T (*n* = 5), ^18^F-PSMA-1007 (*n* = 32), ^18^F-siPSMA-14 (*n* = 9), or ^18^F-PSMA-rhPSMA-7/-7.3 (*n* = 3) according to PSMA-PET/CT imaging protocols [8]. In all centers, two readers were assigned for the interpretation of the imaging findings. Please see Supplementary Table 1 for a detailed description of the imaging protocols of all centers.

### Treatment

The treating radiation oncologists were responsible for the clinical management decisions according to the basis of standards of care at the time of treatment at each respective institution based on the decisions of multidisciplinary tumor boards [8]. Based on PSMA-PET/CT findings and individual patients’ risk factors, treatment has been individualized in terms of sRT region (prostatic fossa ± pelvic lymph nodes), sRT dose as well as inclusion and duration of androgen deprivation therapy (ADT). All patients received intensity-modulated and image-guided sRT to the prostatic fossa and boost irradiation of potentially existent local recurrences dependent on institutional clinical practice. Please see Supplementary Table 2 for a detailed description of the sRT protocols of all centers.

### Follow-up

According to each medical center’s clinical practice, patients underwent follow-up assessments with serum PSA testing at regular intervals and re-staging with medical imaging in terms of biochemical relapse [8]. Please see Supplementary Table 3 for a detailed description of the follow-up protocols of all centers.

### Statistical analysis

The primary study endpoint was biochemical recurrence-free survival, which was defined as PSA nadir after sRT + 0.2 ng/ml or death of any cause. Following BR after sRT, re-staging was performed with imaging (PSMA-PET/CT or CT scans with bone scintigraphy). For the graphical representation, the respective parameters were analyzed by Kaplan–Meier survival curve compared by log-rank test. Uni- and multivariate Cox regression analyses were performed to assess the impact of the different variables on BR. The multivariate Cox regression analysis included significant variables in univariate analysis. In addition, a multivariate logistic regression (LR) analysis was performed to assess the impact of clinical parameters as well as the PET tracers on the detection rate of PSMA-PET/CT imaging after prostatectomy. A two-sided *p* value < 0.05 was considered as statistically significant. The statistical analysis was conducted using SPSS software version: 28.0.1.1. (IBM, USA).

## Results

### Baseline patient and treatment characteristics

The baseline patient and treatment characteristics of the total cohort are listed in Tables 1 and 2. For the total cohort, 97/273 (35.5%) patients had extracapsular disease and 54/273 (19.8%) patients had seminal vesicle invasion in surgery. The median PSA value before sRT was 0.15 (IQR: 0.1–0.18) ng/ml. ADT was given to 36/273 (13.2%) patients. The most frequently applied equivalent dose in 2 Gy per fraction (EQD2, α/β = 1.6 Gy [9]) to the prostatic fossa was 66–70 Gy (*n* = 143/273, 52.4%). SRT to lymph nodes and to elective pelvic lymphatics was delivered in 20/273 (7.3%) and 87/273 (31.9%) patients, respectively.

**Table 1:** Baseline patient characteristics

	Total cohort
Number of patients, *n*	273
Median age at sRT (IQR)	69 (63–74)
Median PSA value before sRT in ng/ml (IQR)	0.15 (1–1.8)
Extracapsular disease in surgery, *n* (%)	
Yes	97 (35.5)
No	162 (59.4)
Unknown	14 (5.1)
Seminal vesicle invasion in surgery, *n* (%)	
Yes	54 (19.8)
No	205 (75.1)
Unknown	14 (5.1)
Resection status in surgery, *n* (%)	
R0	170 (62.3)
R1	85 (31.1)
R2	2 (0.7)
Rx	4 (1.5)
Unknown	12 (4.4)
ISUP grade in surgery, *n* (%)	
1 + 2	95 (34.8)
3	91 (33.3)
4	35 (12.8)
5	50 (18.3)
Unknown	2 (0.7)
Pelvic lymph nodes in surgery, *n* (%)	
Yes	46 (16.8)
No	188 (68.9)
Unknown	39 (14.3)
PSA persistence after surgery, *n* (%)	
Yes	43 (15.8)
No	224 (82)
Unknown	6 (2.2)
Time gap between surgery and recurrent disease, *n* (%)	
≤ 1 year	98 (35.9)
> 1 year	158 (57.9)
Unknown	17 (6.2)
Time gap between PET/CT and sRT, *n* (%)	
≤ 3 months	154 (56.4)
3–6 months	38(13.9)
> 6 months	10 (3.7)
Unknown	71 (26)
Local recurrence on PSMA-PET/CT, *n* (%)	
Yes	81 (29.7)
No	192 (70.3)
Pelvic lymph nodes on PSMA-PET/CT, *n* (%)	
Yes	49(18)
No	224 (82)

*IQR* interquartile range, *ISUP* International Society of Urological Pathology, *PSA* prostate-specific antigen, *sRT* salvage radiotherapy, *PSMA-PET* prostate-specific membrane antigen positron emission tomography

**Table 2:** Treatment characteristics

	Total cohort
Number of patients, *n*	273
Dose* to the prostatic fossa, *n* (%)	
< 66 Gy	31 (11.4)
66–70 Gy	144 (52.7)
> 70 Gy	93 (34.1)
Unknown	5 (1.8)
sRT to elective pelvic lymphatics, *n* (%)	
Yes	82 (30)
No	5 (1.8)
Unknown	186 (68.1)
Dose* to elective pelvic lymphatics, *n* (%)	
≤ 50 Gy	38 (14)
> 50 Gy	17 (6.2)
Unknown	218 (79.9)
sRT to PET/CT positive pelvic lymph nodes, *n* (%)	
Yes	55 (20.1)
No	218 (79.9)
Dose* to PET/CT positive pelvic lymph nodes, *n* (%)	
≤ 50 Gy	6 (2.2)
50–60 Gy	15 (5.4)
> 60 Gy	13 (4.8)
Unknown	239 (87.5)
ADT, *n* (%)	
Yes	64 (23.4)
No	209 (76.6)
Duration of ADT, *n* (%)	
< 6 months	11 (4)
6–12 months	19 (7)
12–24 months	12 (4.4)
> 24 months	6 (2.2)
Unknown	225 (82.4)

*sRT* salvage radiotherapy, *PET* positron emission tomography, *ADT* androgen deprivation therapy

^*^Dose is given in equivalent dose 2 Gy (EQD2, α/β = 1.6 Gy, reference 16), PSMA-PET1: PSMA-PET/CT scan prior to sRT

### Detection rate

For the entire cohort, 118/273 (43.2%) patients had recurrent disease detected with PSMA PET scan before sRT. More specifically, 78/273 (28.6%) patients had local recurrence and 48/273 (17.6%) nodal recurrence, respectively. However, in multivariate LR, none of the clinical parameters had statistically significant impact on the detection rate (Supplementary Table 4), whereas patients with ^68^ Ga-PSMA-11 had a significant lower detection rate compared to patients with ^18^F-PSMA-1007 (OR = 2.204, 95% CI: 1.444–3.363, *p* < 0.001).

### Outcome

**Fig. 1:**
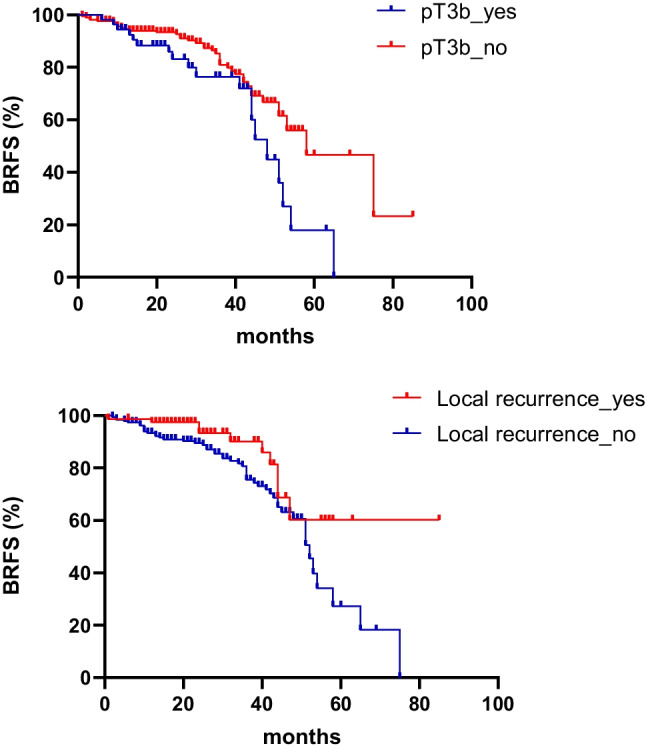
Kaplan–Meier curves for BRFS. Kaplan–Meier curves for BRFS are shown with the risk factors pT3b status in surgery specimen (*p* = 0.027) and with the presence of local recurrence on PET/CT (*p* = 0.035). Statistical comparison was performed with log-rank test

**Fig. 2:**
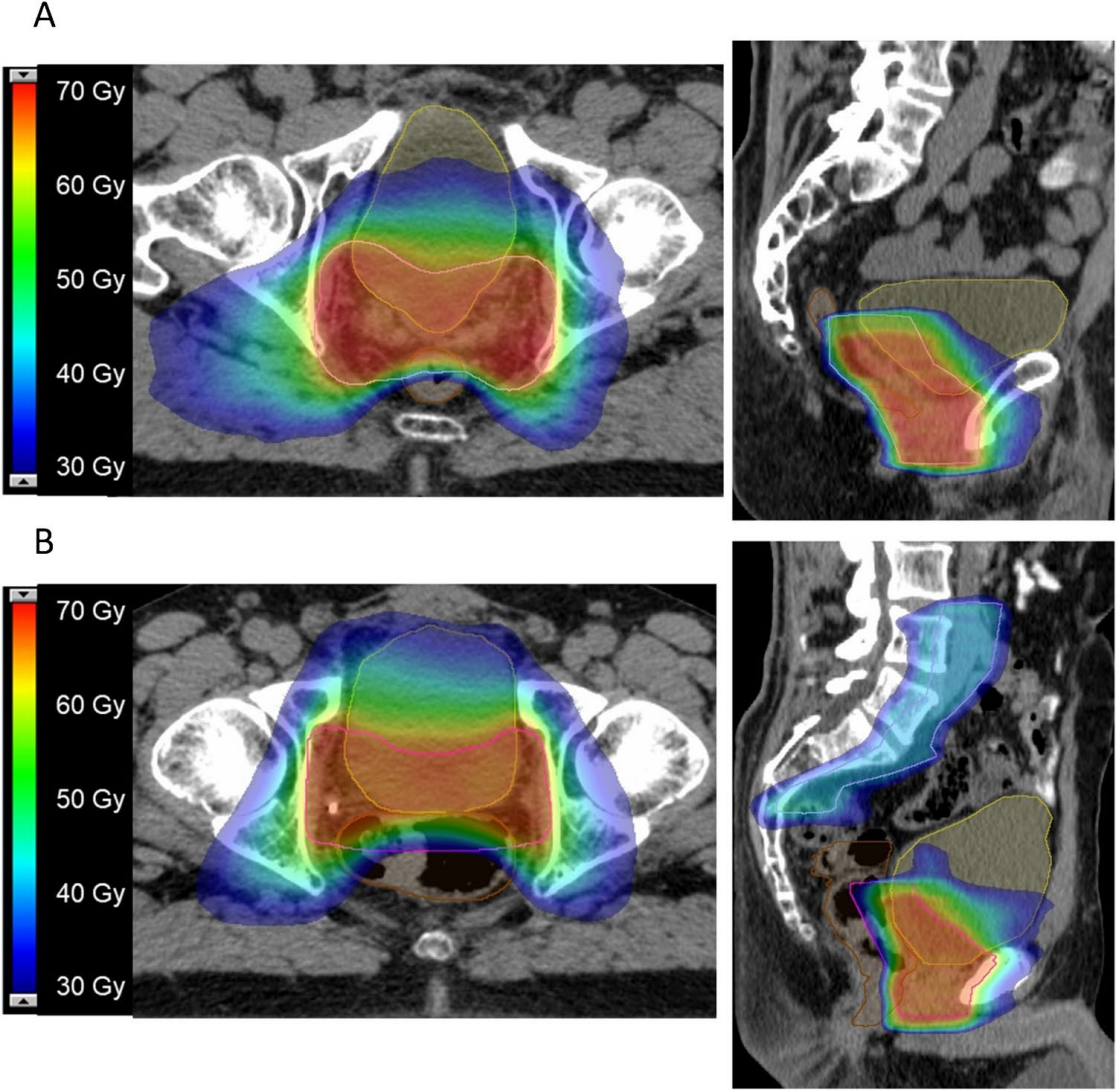
Case reports. This figure shows the treatment plans of two exemplary patients on axial and sagittal planning CT slides. The planning target volume is shown in pink, the rectum in brown, and the bladder in yellow. Radiotherapy isodoses are shown in colorwash according to the legend. Patient A: the patient presented with the following clinical characteristics after definitive prostatecomy: pT2b pN0 (0/26), L0 V0 Pn1 R1, ISUP 3, iPSA 8.5 ng/ml. Before sRT, mpMRI and PSMA-PET did not show any suspicious lesions within or outside the prostate bed. PSA prior to sRT was 0.18 ng/ml. sRT was delivedered to the prostate bed in 1.8 Gy per fraction up to 66.6 Gy. At 44 months of follow-up, no recurrent disease could be observed. Patient B: the patient presented with the following clinical characteristics after definitive prostatectomy: pT3b pN1 (3/25), L1 V0 Pn1 R1, ISUP 3, IPSA 13.8 ng/ml. Before sRT, mpMRI and PSMA-PET did not show any suscipious lesions within or outside the prostate bed. sRT was deleivered to the prostate bed in 1.8 Gy per fraction up to 66.6 Gy. Elective pelvis were treated with 1.8 Gy per fraction up to 45 Gy. Regions with positive lymph nodes were escalated with boost of 1.8 Gy per fraction up to 54 Gy. Androgen deprivation therapy was given for 6 months. After 31 months, the patient experienced a biochemical recurrence. PSMA-PET/CT imaging revealed a bone metastasis in the breast vertebrae 7

After a median follow-up time of 31.1 months (IQR: 20–44), 60/273 (22%) patients had biochemical recurrence. One patient deceased due to progressive PCa 59 months after BR. At 2 and 3 years, the estimated rates of BRFS were 90.1% and 79.2%, respectively. The detailed results of the Cox regression analyses are listed in Table 3. The presence of seminal vesicle invasion in surgery (HR = 1.945, 95% CI: 1.114–3.393, *p* = 0.019) and local recurrences in PET/CT (HR = 0.488, 95% CI: 0.246–0.945, *p* = 0.039) had a significant impact on BRFS in univariate and multivariate analysis. Please see Fig. 1 for the Kaplan–Meier representation of both risk factors. Two representative patient cases are presented in Fig. 2. There was no significant difference in BRFS (HR = 0.554, 95% CI: 0.204–1.504, *p* = 0.246) between patients which were staged with the ^18^F-PSMA-1007 or ^68^ Ga-PSMA-11 tracer in Cox regression analysis.

**Table 3:** Univariate and multivariate Cox regression analysis for BR

Variable	Univariate HR (95% Cl)	*P* value	Multivariate HR (95% Cl)	*P* value
pT3a status in surgery (yes vs. no)	0.96 (0.561–1.645)	0.893		
pT3b status in surgery (yes vs. no)	**1.945 (1.114–3.393)**	**0.017**	**1.944 (1.113–3.394)**	**0.019**
pN status in surgery (yes vs. no)	1.788 (0.967–3.306)	0.064		
Resection status (R0 vs. R1 + R2 + Rx)	1.303 (0.763–2.25)	0.333		
ISUP score (1 + 2 vs. 3 + 4 + 5)	1.865 (0.985–3.531)	0.056		
PSA persistence after surgery (yes vs. no)	1.488 (0.769–2.878)	0.238		
Time gap from surgery to sRT (≤ 1 year vs. > 1 year)	0.924 (0.542–1.577)	0.773		
Local failure in PSMA-PET/CT (yes vs. no)	**0.488 (0.246–0.945)**	**0.033**	**0.459 (0.224–0.940)**	**0.039**
Nodal failure in PSMA-PET/CT (yes vs. no)	1.345 (0.582–2.078)	0.769		
sRT dose* to fossa (≤ 66 Gy vs. > 66 Gy)	0.853 (0.303–2.387)	0.758		
ADT admission (yes vs. no)	1.678 (0.969–2.905)	0.064		S

Significant variables in uni and multivariate analyses are presented in bold

*HR* hazard ratio, *CI* confidence interval, *ISUP* International Society of Urological Pathology, *PSA* prostate-specific antigen, *sRT* salvage radiotherapy, *PSMA-PET/CT* prostate-specific membrane antigen positron emission tomography, *ADT* androgen deprivation therapy

^*^Dose is given in equivalent dose 2 Gy (EQD2, α/β = 1.6 Gy, reference 16). Local failure: failure in the prostatic fossa, nodal failure: failure in pelvic lymph nodes

### Recurrence patterns

In 48/60 (80%) patients with PSA relapse after sRT, a second PSMA-PET/CT scan was conducted and information on recurrence patterns was available in 16 patients. Local relapse was detected in three patients and four patients had positive pelvic lymph nodes. Distant metastases were observed in 10 patients, and of these, 3 patients had lymph nodes outside the pelvis and 7 had bone metastases. Recurrent disease after sRT was localized outside the RT field in 15 of the 16 patients (93.8%).

## Discussion

PSMA-PET/CT imaging is increasingly used to guide sRT after prostatectomy mostly at PSA levels > 0.2 ng/ml. However, several studies suggested a significant improvement in BRFS or MFS when sRT is performed at low (< 0.2 ng/ml) PSA levels [2]. On the contrary, the detection rate of PSMA-PET/CT imaging was reported to be low in this patient population [6, 7]. In our study, we retrospectively included 273 patients with PSA ≤ 0.20 ng/ml and observed a detection rate of 43.2%. Gupta et al. [10] examined a subgroup of patients with prior prostatectomy and PSA levels ≤ 0.20 ng/ml at biochemical relapse. The authors demonstrated a detection rate of 46%. According to a meta-analysis [11], ^68^ Ga-PSMA-11 PET/CT detection rates for patients with PSA levels of < 0.2 ng/ml after prostatectomy ranged from 11.3 to 58.3%. Thus, the detection rate in our study is comparable to other studies, and it can be assumed that approximately one-third of the patients in this patient population might receive a change in sRT treatment concept based on PSMA-PET/CT findings, like a RT boost to the prostatic fossa, inclusion of the pelvic lymphatics, or administration of ADT. In our study, ADT was given to 13% of patients and elective pelvic lymphatics were treated in 32% of patients. Whether a treatment individualization based on PET/CT findings impacts the oncologic outcome in patients with PSA ≤ 0.20 ng/ml was not examined in current literature.

Consequently, the main aim of this study was to assess the outcome after PSMA-PET/CT-based sRT in terms of BRFS reported as 90.1% and 79.2% two and three years after sRT. Several retrospective studies reported the BRFS after early sRT in the pre-PSMA-PET/CT era. Abhugarib et al. [3] included 657 patients and reported a 2-year BRFS of 85%. Similar results were observed by Tendulkar et al. [2] with an estimated BRFS of 82% two years after sRT. A direct comparison of the BRFS rates between our study and the two others is hampered by different patient cohorts, follow-up protocols, and definition of BR. However, our results suggest that the induction of PSMA-PET/CT for sRT guidance might provide a possible benefit in this dedicated group of patients, which should be evaluated further. The currently ongoing, prospective PSMA-SRT study evaluates the success rate of sRT for recurrence of PCa after prostatectomy with and without planning based on PSMA-PET/CT [12], and this study includes patients with PSA > 0.1 ng/ml.

In this study, two clinical parameters (namely, seminal vesicle invasion in surgery and local recurrences on PET/CT imaging) had a significant impact on BR in multivariate analysis. Interestingly, local recurrent disease on PET/CT had a negative influence on BR (HR = 0.488). At initial observation, this finding is unexpected, as one would assume that macroscopic local disease could lead to an unfavorable BRFS. However, this might be interpreted by the fact that patients with such low PSA values and only local uptake in PET have a very low risk for distant metastatic disease. Thus, it is very likely that the recurrent prostate cancer in this patient group is cured by sRT to the prostatic fossa. Future studies should address the optimal sRT dose to the fossa [13] as well as to the local recurrence in PET/CT and whether admission of ADT is needed in this scenario [14]. Whether the used PSMA tracer has an impact on BRFS after sRT is not answered yet. In our study, patients with the ^18^F-PSMA-1007 tracer had a significant better detection rate compared to patients with the ^68^ Ga-PSMA-11 tracer. However, this was not translated into a significant better BRFS. Future studies with more patients and longer follow-up should assess this question. In addition, a study by Spohn et al. proposed that the SUVmax value with the local recurrent disease on PSMA-PET/CT images might be a predictive factor for BRFS after sRT [15].

Information on recurrence patterns on PSMA-PET/CT was available in 16 out of 60 patients with PSA relapse after sRT. Most patients had distant relapse and only one patient had recurrent disease inside the RT field, suggesting that implementation of PSMA-PET/CT information for sRT has a benefit in the local control of the disease.

Despite its multicenter character, our study has some limitations. First, due to its retrospective nature, PSMA-PET/CT/CT, sRT, and follow-up protocols were not consistent within all study centers. Moreover, central PET/CT imaging and pathology review, which ensures consistency between the different reports, has not been carried out. Third, the number of patients included in this study is relatively small as staging using PSMA-PET/CT is not frequently used in patients with PSA < 0.2 ng/ml. In addition, as the PSMA-PET/CT has been recently implemented in clinical practice, follow-up time in our study is relatively short. Finally, although our primary endpoint—biochemical recurrence—is commonly used in studies on sRT after surgery, it is not a surrogate endpoint for overall survival or death of prostate cancer [16].

## Conclusion

This multicenter analysis included patients with PSMA-PET/CT-guided sRT at low PSA levels after primary prostatectomy (< 0.2 ng/ml). Our results demonstrate very promising BRFS rates as well as a low number of in-sRT field recurrences, suggesting that implementation of PSMA-PET/CT imaging for sRT guidance might be of benefit for this patient cohort. In addition, the presence of seminal vesicle invasion in surgery specimen and the absence of local recurrence in PET/CT were presented as new risk factors for BRFS after PSMA-guided sRT. Further prospective studies are warranted to validate our findings.

## Supplementary information

Below is the link to the electronic supplementary material.

Supplementary file1 (DOCX 18 KB)

Supplementary file2 (DOCX 20 KB)

Supplementary file3 (DOCX 15 KB)

Supplementary file4 (DOCX 15 KB)

## Data Availability

The datasets generated during and/or analysed during the current study are not publicly available due to GDPR reasons but are available from the corresponding author on reasonable request and under adherence to GDPR and ethic requirements.
