# Supplementary material for: PSMA-PET/CT-guided salvage radiotherapy in recurrent or persistent prostate cancer and PSA < 0.2 ng/ml
Source: Eur J Nucl Med Mol Imaging. 2023 Mar 11;50(8):2529–36. doi: 10.1007/s00259-023-06185-5 (PMC10250454; doi:10.1007/s00259-023-06185-5)
Supplement: Supplementary file 1 — Supplementary file1 (DOCX 18 KB) [file 259_2023_6185_MOESM1_ESM.docx]

**Supplementary Material – Table 1. PSMA PET Imaging protocols**

| **Center** | **PET/CT or PET/MR system(s)** | **Tracer(s)** | **Post injection time (hours)** |
| --- | --- | --- | --- |
| Freiburg, Germany | GEMINI TF TOF 64, GEMINI TF 16 Big Bore and Vereos (all from Philips, The Netherlands) | 68Ga-PSMA-11  18F-PSMA-1007 | 68Ga-PSMA-11: 1  18F-PSMA-1007: 2 |
| Limassol, Cyprus | Discovery IQ PET/CT system (GE, United States) | 18F-PSMA-1007 | 2 |
| Sydney, Australia | Biograph PET/64-slice CT (Siemens, Germany)  Ingenuity/64-slice CT (Phillips, The Netherlands) | 68Ga-PSMA-11 | >1 |
| Hannover, Germany | Biograph PET/128-slice CT (Siemens, Germany) | 68Ga-PSMA-11 | 1 |
| LMU Munich, Germany | Siemens Biograph 64 (Siemens, Germany)  Discovery 690 (GE, United States) | 68Ga-PSMA-11  18F-PSMA-1007 | 1 |
| TUM Munich, Germany | Biograph mCT / 128 slice CT  Biograph mMR, (both Siemens, Germany) | 68Ga-PSMA-11  18F-PSMA-1007  18F-rhPSMA-7  18F-rhPSMA-7.3 | 68Ga-PSMA-11: 1  18F-PSMA-1007: 1.5  18F-rhPSMA-7: 1  18F-rhPSMA-7.3: 1 |
| Bologna, Italy | Discovery MI and Discovery 710 (both GE, United States) | 68Ga-PSMA-11 | 1 |
| Zürich, Switzerland | PET/MR: SIGNA PET/MR. PET/CT: Discovery MI and Discovery VCT 690 (all GE, United States) | 68Ga-PSMA-11 | 1 |
| Ulm, Germany | Biograph mCT / 40 slice CT  Biograph mMR (both Siemens, Germany) | 68Ga-PSMA-11  18F-siPSMA-14 | 68Ga-PSMA-11: 1  18F-siPSMA-14: 1.5 |
| Bern University Hospital, Inselspital, Berne, Switzerland | Biograph Vision Quarda (Siemens, Germany)  Siemens Biograph 64 (Siemens, Germany | 68Ga-PSMA-11  18F-PSMA-1007 | 68Ga-PSMA-11: 1  18F-PSMA-1007: 2 |
| Heidelberg, Germany | Biograph mCT Flow scanner  Biograph 6 PET/CT scanner  Biograph 20 mCT scanner  (all Siemens, Germany) | 68Ga-PSMA-11  18F-PSMA-1007 | 68Ga-PSMA-11: 1  18F-siPSMA-14: 1.5 |
