# Supplementary material for: PSMA-PET/CT-guided salvage radiotherapy in recurrent or persistent prostate cancer and PSA < 0.2 ng/ml
Source: Eur J Nucl Med Mol Imaging. 2023 Mar 11;50(8):2529–36. doi: 10.1007/s00259-023-06185-5 (PMC10250454; doi:10.1007/s00259-023-06185-5)
Supplement: Supplementary file 2 — Supplementary file2 (DOCX 20 KB) [file 259_2023_6185_MOESM2_ESM.docx]

**Supplementary Material – Table 2. Salvage RT protocols**

| **Center** | **Dose to fossa (EQD2, α/β=1.6 Gy)** | **Indication (and field) for sRT to elective pelvic lymphatics** | **Dose to elective pelvic lymphatics and PET positive pelvic LN (EQD2, α/β=1.6 Gy)** | **Indication (and duration) of androgen deprivation therapy** |
| --- | --- | --- | --- | --- |
| Freiburg, Germany | Dose to fossa (R0): 64 Gy  Dose to fossa (R1): 66-68 Gy  Dose to local recurrence in PET: 68-72 Gy | pN+ status in surgery (whole-pelvis)  Positive pelvic LNs in PSMA- PET/CT (whole-pelvis) | Elective pelvic lymphatics: 42.5-47.6 Gy  PET positive pelvic LN 50-60 Gy | pN+ status (0-24 months)  PSA prior to sRT >0.7 ng/ml (6 months)  Positive pelvic LNs in PET (6-24 months)  + individual decision (e.g depending on comorbidities) |
| Limassol, Cyprus | Dose to fossa (R1): 71.2 Gy (hypofractionated physical dose: 62.5Gy in 2.5Gy)  Dose to fossa (R0): 66 Gy  Dose to local recurrence in PET: 70-72 Gy | pN+ status in surgery (whole-pelvis)  Positive pelvic LNs in PSMA-PET/CT (whole-pelvis) | Elective pelvic lymphatics: 50Gy  PET positive pelvic LN 66.7Gy (physical dose: 60Gy in 2.4Gy) | pN+ status (<18 months)  pT3 or GS 8-10 (<12 months)  Positive pelvic LNs in PET (<12 months)  + individual decision (e.g depending on comorbidities) |
| Sydney, Australia | Local recurrence in PET: 70 Gy to local recurrence and 64 Gy to the rest of the fossa  No local recurrence in PET: 68 Gy to the entire fossa | pN+ status in surgery (whole-pelvis)  Positive pelvic LNs in PSMA-PET/CT (whole-pelvis) | Elective pelvic lymphatics (+/-) including the PET positive nodes: 54-56 Gy | Positive pelvic LNs in PET (6 months)  + individual decision (e.g depending on comorbidities) |
| Hannover, Germany | Dose to fossa (R0): 66 Gy  Dose to fossa (R1): 68-72 Gy  Dose to local recurrence in PET: 68-74 Gy | pN+ status in surgery (whole-pelvis)  Positive pelvic LNs in PSMA-PET/CT (whole-pelvis) | Elective pelvic lymphatics: 50 Gy  PET positive pelvic LN: SIB Boost 57.5 Gy in 2.3 Gy | No androgen deprivation therapy was given |
| LMU Munich, Germany | Dose to fossa: 66 Gy  (in case of whole pelvis RT: 64.75 Gy)  Dose to local recurrence: 70 Gy (in case of whole pelvis RT: 72.2 Gy) | pN+ status in surgery (whole- pelvis)  positive pelvic LNs in PSMA-PET/CT (whole-pelvis) | Elective pelvic lymphatics: 46.75 Gy  PET-positive LNs: 64.75 Gy | Positive pelvic LNs in PET (6-24 months)  positive local recurrence in PET (6-24 months)  PSA prior to sRT > 0.7 ng/ml and Gleason 8-10 (6-24 months)  + individual decision (depending on comorbidities, Gleason, PSA prior to sRT) |
| TUM Munich, Germany | Dose to fossa: 68 Gy  Dose to local recurrence in PET: 82.88 Gy (SIB, 76.5 Gy in fractions of 2.25 Gy) | pN+ status in surgery, lymph node dissection with <10 lymph nodes, or risk for lymph node involvement of ≥20% (whole-pelvis)  Positive pelvic LNs in PSMA-PET/CT (whole-pelvis) | Elective pelvic lymphatics: 47.04 Gy (50.4 Gy in fractions of 1.8 Gy)  PET positive pelvic LN: 60.76 Gy (SIB, 58.8 Gy in fractions of 2.1 G) or 65.71 Gy (SIB, 61.6 Gy in fractions of 2.2 Gy) | PSA prior to sRT >0.7 ng/ml (6-24 months)  + individual decision (tumor conference) |
| Bologna, Italy | Dose to fossa: 66-70 Gy  None of the patients had a PET positive recurrence in the fossa | Positive pelvic LNs in PSMA-PET/CT (half/ whole pelvis) | Elective pelvic lymphatics (+/-) including the PET positive nodes:  half-pelvis: 45-50 Gy  whole-pelvis: 45-60 Gy | Positive pelvic LNs in PET (6-24 months)  + individual decision (e.g depending on comorbidities |
| Zürich, Switzerland | Dose to the fossa: 66Gy  Dose to local recurrence: 72-74Gy | Positive pelvic LNs in PSMA-PET/CT (whole pelvis) | Elective pelvic lymphatics 45-50.4Gy. PET positive pelvic LN: SIB Boost 56Gy or SBRT Boost with 2x5Gy (calculated to the 65%-80% Isodose) | Individual decision when:  - cN1 in PSMA-PET  - Initially pT3b/4 and ISUP>4  - Initially pT3b/4 and PSA>0.7 ng/ml  - Initially ≥pT2 and R1  - ADT 6-24 months |
| Ulm, Germany | Dose to fossa: 72 Gy | Positive pelvic LNs in PSMA  PET/CT (whole pelvis) | Elective pelvic lymphatics 51 Gy  PET-positive nodes: 64-66 Gy | Positive pelvic nodes: 2 years ADT  PET/CT-N0: individual decision: PSA >0.7 and or Gleason 8-10 6-24 months |
| Bern University Hospital, Inselspital, Berne, Switzerland | Dose to the fossa: 66Gy  Dose to local recurrence: 72-74Gy | Positive pelvic LNs in PSMA-PET/CT (whole pelvis) | Elective pelvic lymphatics 50.4Gy. PET positive pelvic LN with SIB to 64.4 Gy | ADT for 6 months offered if:    - Pre-SRT PSA > 0.6 ng/ml  - Evidence of Macroscopic disease within prostate bed  - N1 in PSMA PET |
| Heidelberg, Germany | Dose to fossa: 68-70 Gy  Dose to local recurrence in PET: 70-81.8 Gy (depending on site of relapse) | pN+ status in surgery, lymph node dissection with <10 lymph nodes and risk for lymph node involvement of ≥ 20% (whole-pelvis)  Positive pelvic LNs in PSMA-PET/CT (whole-pelvis) | Elective pelvic lymphatics: 43.9 Gy (51 Gy in 34 fractions)  PET positive pelvic LN: SIB Boost 57.8 Gy (61.2 Gy in 34 fractions) | pN+ status (24 months)  Positive pelvic LNs in PET (24 months)  PSA prior to sRT >0.7 ng/ml (6-24 months)  + individual decision (e.g depending on comorbidities) |

Abbreviations: PSMA-PET/CT: prostate-specific membrane antigen positron emission tomography/computed tomography, LN: lymph nodes, SIB: simultaneously integrated boost, PSA: prostate-specific antigen, sRT: salvage radiotherapy, all doses are given in equivalent dose 2 Gy (EQD2, α/β=1.6 Gy, reference 16),
