# Supplementary material for: PSMA-PET/CT-guided salvage radiotherapy in recurrent or persistent prostate cancer and PSA < 0.2 ng/ml
Source: Eur J Nucl Med Mol Imaging. 2023 Mar 11;50(8):2529–36. doi: 10.1007/s00259-023-06185-5 (PMC10250454; doi:10.1007/s00259-023-06185-5)
Supplement: Supplementary file 3 — Supplementary file3 (DOCX 15 KB) [file 259_2023_6185_MOESM3_ESM.docx]

**Supplementary Material – Table 3. Follow-up concepts**

| **Center** | **Frequency of PSA tests** | **Indication for re-staging with diagnostic imaging after sRT** | **Imaging used for re-staging after sRT** |
| --- | --- | --- | --- |
| Freiburg, Germany | Every 3 months in the first 2 years after sRT. Then every 6 months for the next three years and then 6-12 months. | Biochemical recurrence (as defined in the manuscript)  In case of one negative PSMA-PET/CT after sRT: at PSA levels of approximately 1 ng/ml or yearly (according to the treating physician) | Preferably, PSMA-PET/CT scans |
| Limassol, Cyprus | Every 3 months in the first 2 years after sRT. Then every 6 months for the next year, yearly thereafter. | Biochemical recurrence (as defined in the manuscript)  No use of imaging modalities unless PSA rise | Preferably, PSMA-PET/CT scans |
| Sydney, Australia | Every 6 months | Biochemical recurrence (defined as PSA >0.2 ng/ml) | PSMA-PET/CT scans |
| Hannover, Germany | Every 3 months for five years | Biochemical recurrence (as defined in the manuscript) | Preferably, PSMA-PET/CT scans |
| LMU Munich, Germany | Every 3 months | Biochemical recurrence (as defined in the manuscript)  No use of imaging modalities unless PSA rise | Preferably, PSMA-PET/CT scans |
| TU Munich, Germany | Every 3 months in the first 2 years after sRT. Then every 6 months for the next three years and then 6-12 months. | Biochemical recurrence (as defined in the manuscript) | Preferably, PSMA-PET/CT scans |
| Bologna, Italy | Every 3 months in the first 2 years after sRT. Then every 6 months for the next year, yearly thereafter. | Biochemical recurrence (as defined in the manuscript)  No use of imaging modalities unless PSA rise | Preferably, PSMA-PET/CT scans |
| Zürich, Switzerland | Every 3 months | Biochemical recurrence (as defined in the manuscript) | Preferably, PSMA-PET/CT or -PET/MRI scans |
| Ulm, Germany | Every 3 months in the first 2 years after sRT. Then every 3-12 months for the next five years. | Biochemical recurrence (as defined in the manuscript) | Preferably, PSMA-PET/CT scans |
| Bern University Hospital, Inselspital, Berne, Switzerland | Every 3 months in first year after SRT then every 6 months for 3 years and once per year till 5 years after SRT. | Biochemical recurrence (as defined in the manuscript) | Preferably, PSMA-PET/CT scans |
| Heidelberg, Germany | Every 3 months in the first 2 years after sRT. Then every 6 months for the next three years and then 6-12 months. | Biochemical recurrence (as defined in the manuscript) | Preferably, PSMA-PET/CT scans |

Abbreviations: sRT: salvage radiotherapy, PSA: prostate-specific antigen, PSMA: prostate-specific membrane antigen
