# Supplementary material for: PSMA-PET/CT-guided salvage radiotherapy in recurrent or persistent prostate cancer and PSA < 0.2 ng/ml
Source: Eur J Nucl Med Mol Imaging. 2023 Mar 11;50(8):2529–36. doi: 10.1007/s00259-023-06185-5 (PMC10250454; doi:10.1007/s00259-023-06185-5)
Supplement: Supplementary file 4 — Supplementary file4 (DOCX 15 KB) [file 259_2023_6185_MOESM4_ESM.docx]

**Supplementary Material – Table 4. Impact on clinical characteristics on detection rate**

|  |  | 95%CI for OR | |  |
| --- | --- | --- | --- | --- |
|  | OR | lower | upper | p - value |
| pT3a status in surgery (yes vs no) | 1.002 | 0.527 | 1.907 | 0.994 |
| pT3b status in surgery (yes vs no) | 2.196 | 0.978 | 4.931 | 0.057 |
| Resection status (R0 vs R1+R2+Rx) | 0.927 | 0.498 | 1.726 | 0.811 |
| ISUP score (1+2 vs 3+4+5) | 0.836 | 0.451 | 1.547 | 0.568 |
| PSA persistence after surgery (yes vs no) | 1.167 | 0.494 | 2.757 | 0.726 |
| Time gap from surgery to sRT (≤1 year vs >1 year) | 1.664 | 0.887 | 3.125 | 0.113 |
| pN status in surgery (yes vs no) | 0.702 | 0.333 | 1.479 | 0.352 |

Abbreviations: OR: odds ratio, CI: confidence interval, PSA: prostate-specific antigen, sRT: salvage radiotherapy
